# Impression Cytology and *In Vivo* Confocal Microscopy of Lip Mucosa Compared With Labial Gland Biopsy and Classification Criteria In Patients With Clinically Suspected Primary Sjögren’s Syndrome

**DOI:** 10.3389/fimmu.2022.829320

**Published:** 2022-05-02

**Authors:** Ran Hao, Ziyuan Liu, Yilin Chou, Yuexin Wang, Xiaotong Ren, Xiaodan Jiang, Xuemin Li

**Affiliations:** ^1^ Department of Ophthalmology, Peking University Third Hospital, Beijing, China; ^2^ Beijing Key Laboratory of Restoration of Damaged Ocular Nerve, Peking University Third Hospital, Beijing, China

**Keywords:** impression cytology, *in vivo* confocal microscopy, labial gland biopsies, lip mucosa, primary Sjögren’s syndrome

## Abstract

**Purpose:**

The study assessed the validity of impression cytology (IC) and *in vivo* confocal microscopy (IVCM) of lip mucosa compared with labial gland biopsy, anti-Sjögren’s syndrome A (SSA)/Ro antibody status, and classification criteria in suspected primary Sjögren’s syndrome (pSS) patients.

**Methods:**

Clinically suspected pSS patients (n = 201) were enrolled consecutively and were divided into pSS (n = 56) and control (n = 145, only with dryness) groups according to the American College of Rheumatology-European League Against Rheumatism (ACR-EULAR) criteria. All patients underwent lip mucosa IC (inflammatory cell density) and IVCM (epithelium/intrinsic layer thickness and labial gland density/diameter) analyses. The associations between IC/IVCM parameters and clinical/laboratory results were analyzed.

**Results:**

The absolute agreement between positive lip mucosal IC (≥50 cells/4 mm^2^) and the ACR-EULAR criteria (94.5%)/labial gland biopsy (95.5%) was good, with sensitivities of 82.1 and 85.2%, respectively, and a specificity of 99.3%. Compared with controls, IVCM revealed significant lip mucosal atrophy and glandular decreases in the pSS group (all *P* = 0.000). The sensitivities for diagnosing pSS corresponding to a lamina propria thickness ≤128 μm and a gland diameter ≤114 μm were 85.7 and 89.3%; the specificities were 90.3 and 95.9%, respectively. A combination of positive IC/IVCM and anti-SSA/Ro antibody results showed a high predictive value for diagnosing pSS.

**Conclusions:**

IC and IVCM could detect distinctive cellular and morphological changes in the lip mucosa of patients with pSS. These noninvasive and easy-to-perform examinations may be an alternative to labial gland biopsy for diagnosing pSS.

## 1 Introduction

Primary Sjögren’s syndrome (pSS) is a complex, disabling, autoimmune disease with diverse phenotypes, variable outcomes, and a male-to-female dominance of 1:9 (1, 2). The clinical spectrum varies from chronic inflammation of exocrine glands to an aggressive multisystem disease (2–7), leading to organ damage, even failure, and non-Hodgkin’s lymphoma in 10–20% and 5–10% of patients, respectively, within the first 10–15 years (4, 7–10). Currently, the classification of pSS is based on a combination of clinical dryness manifestations, histological, and serological parameters (3). However, those criteria may not be sensitive enough to early disease and are often met in the advanced stage of the disorder (accompanied by severe complications and terrible life quality) (3). Therefore, accurate and early diagnosis of pSS plays a major role in disease management.

Clinically, the Sjögren’s syndrome dry eye patients are always suffering from severe xerophthalmia and ocular surface injury, resulting in therapeutic difficulties and even blindness (11, 12). With the help of *in vivo* confocal microscopy (IVCM), the characteristic density and morphology alterations of corneal nerves and dendritic cells (DCs) in Sjögren’s syndrome dry eye have been identified and provide a better insight into the pathogenesis of clinical manifestations (13). Salivary gland biopsy is a hallmark of pSS and has a crucial position in routine classification criteria (2, 3). However, it is not frequently performed as it is an invasive procedure (14), and follow-up biopsies are not feasible (15). The morphological changes of the oral mucosa could also be captured by IVCM in real-time and showed excellent correlation with histopathology (16, 17). Another mini-invasive pathological tool is impression cytology (IC), which has been widely used in ocular surface diseases (18–24). IC sampling removes the three most superficial layers of the mucosa by adhesion to cellulose-acetate filters; subsequently, the adhered tissue is stained for histological analysis (25, 26). Compared to the tissue disruption caused by salivary gland biopsies, IC is painless, well-tolerated, and widely available in outpatient clinics (25). Furthermore, samples can be repeatedly obtained within a short period. The microscopic details of living tissues can be observed without the need for biopsies or anesthetics, suggesting the potential value of IC and IVCM in improving prognostics, diagnostics, and patient-stratification of pSS.

Thus, we explored the prognostic and diagnostic value of IC and IVCM in patients with clinically suspected pSS. Furthermore, to clarify whether IC/ICVM can be used as a clinically noninvasive tool to stratify patients and yield insights into the pathology.

## 2 Materials and Methods

### 2.1 Study Design and Participants

Clinically suspected pSS patients (n = 216), complaining of dry eyes and mouth in 18–80-year-old individuals, were consecutively included from January 2017 to March 2020. The exclusion criteria were as follows: (1) oral mucositis, lip surgery or other forms of salivary gland dysfunction (such as induced by radiation) within the previous year, (2) connective tissue disease, (3) acute systemic infection, (4) pregnancy, (5) medication use associated with the Sicca syndrome, and (6) smoking and other forms of tobacco use. Finally, 201 participants were included in this study. According to the American College of Rheumatology-European League Against Rheumatism (ACR-EULAR) criteria (2), all participants underwent labial gland biopsy, serum anti-Sjögren’s syndrome A (SSA)/Ro antibody testing, Schirmer’s test (ST), ocular surface staining (OSS), and unstimulated whole saliva flow (UWS) determinations, and were divided into the pSS group (n = 56) and control group (n = 145). The control group consisted of subjects with just dryness and no Sjögren’s syndrome. All participants underwent lip mucosal IC and IVCM as part of their diagnostic work-up. The study was conducted according to the principles of the Declaration of Helsinki and approved by the Human Research and Ethics Committee (No. M2019236). Written informed consent was obtained from each study participant.

### 2.2 IC of the Lip Mucosa

The samples were collected with a sterilized cellulose acetate filter membrane disc (Millipore, Barueri, SP, Brazil), which was cut into circles of 4.00 mm in diameter. IC samples were obtained from the left, middle, and right regions of the lower lip mucosa, using the technique described previously (25). Briefly, patients gently pulled their lower lips to completely expose the internal lip mucosa. Without the need for anesthesia, a filter material (rough surface toward the globe) was applied to the internal lip mucosa using toothless forceps and was gently pressed with a glass rod. The filter membrane was left in place for 20 s and then gently peeled from the oral mucosa surface. The first to third most superficial epithelial layers could be removed by the application of the filter material (26). The same regions were sampled thrice, and the last three IC samples from the corresponding regions were rapidly dehydrated in 95% ethanol and stored at 4°C. After washing in distilled water and phosphate buffer solution, the specimens were stained with hematoxylin and eosin (H&E), and observed using a light microscope (Zeiss, Oberkochen, Germany). Since the IC can remove cell layers, this was examined after oral IVCM and before labial gland biopsy.

### 2.3 Lip Mucosa IVCM

**Figure 1:**
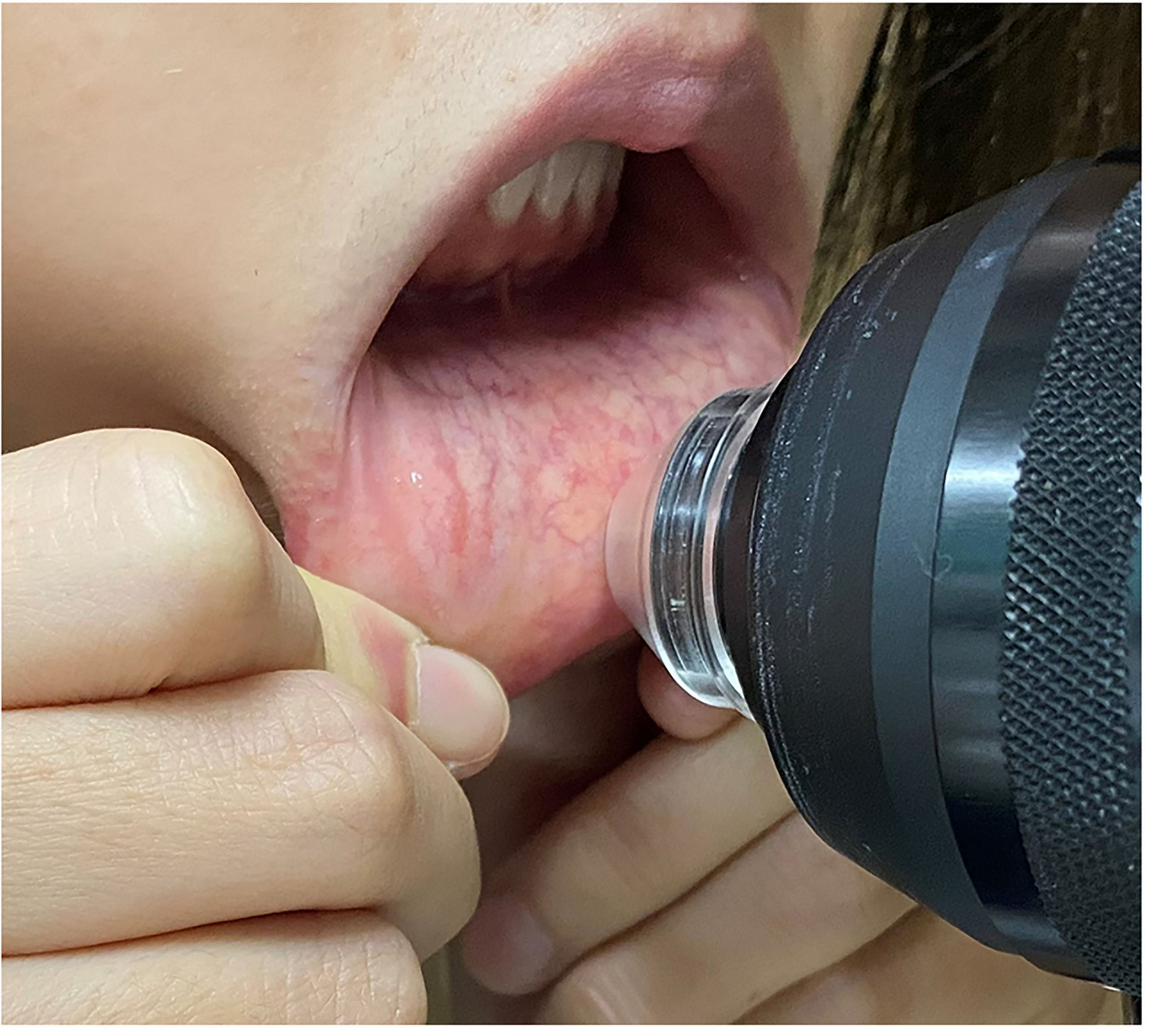
A confocal laser-scanning microscope (HRT II RCM, Heidelberg Engineering, Heidelberg, Germany) to use in the labial mucosa.

A digital confocal laser-scanning microscope (HRT II RCM, Heidelberg Engineering, Heidelberg, Germany) was used to observe the microstructure of the labial mucosa. Two-dimensional pictures were obtained, with a definition of 384 × 384 pixels over an area of 400 µm × 400 µm, a lateral spatial resolution of 0.5 µm, and a depth resolution of 1–2 µm. Without the need for anesthesia, patients gently pulled their lower lips to completely expose the internal lip mucosa (**Figure 1**). The objective needs to touch the inner lip surface to obtain images. The disposable probe is tiny so that we can control the detection sites. For each patient, vertical stacks of more than 100 images, recording the epithelium through to the level of the lamina propria, were captured for each patient. Five good quality images from five different positions were chosen for calculating the average thickness of the lip mucosa. A total of five images with the best focus and contrast, without motion or folds, were chosen for density and morphology analysis using semiautomatic software (Image J; National Institutes of Health, Bethesda, MD, USA); an average of five measurements was recorded. The thicknesses of the epithelium layers (between the appearance and disappearance of the epithelial cells), intrinsic layers (between the appearance and disappearance of the labial glands) and labial gland density/diameter were evaluated by two blinded observers with an intraclass correlation coefficient range of 0.908 to 0.964.

### 2.4 Ocular and Systemic Assessments

The ocular surface disease index (OSDI), ST, tear meniscus height (TMH), tear film breakup time (TBUT), and OSS score were determined using a standard protocol (11). All labial gland biopsies were performed by an experienced dentist after obtaining surgical informed consent. Labial minor salivary gland biopsies were performed by everting the lower lip to visualize the minor salivary glands located just below the mucosa. A 1:100,000 1% lidocaine:epinephrine solution (0.5–1.0 ml) was carefully injected into the submucosa and a 1.0–1.5 cm linear incision was made into the mucosa, parallel to the vermilion border. Blunt dissection was performed on a plane perpendicular to the mucosal incision and parallel to the direction of the sensory nerve fibers; 4–7 glands were recovered from each patient for definitive histopathological analyses. Finally, the incision was closed with simple interrupted 4–0 chromic sutures. The biopsy results were considered positive if the focus score (defined as the number of mononuclear infiltrates containing ≥50 lymphocytes/4 mm^2^ of glandular tissue) was ≥1 (1, 2). Unstimulated whole saliva flow (UWS) was evaluated using measured saliva production within 15 min. A UWS ≤0.1 ml/min was deemed abnormal (1, 2). Serum levels of anti-SSA/Ro antibodies, anti-Sjögren’s syndrome B (SSB) antibodies, rheumatoid factor (RF), and antinuclear antibody (ANA) were assessed with ELISA tests (27) The parotid glands were scored using a simplified salivary gland ultrasonography (SGUS) scoring system (6). Parenchymal homogeneity in salivary glands was scored 0–3 (0, normal; 1, mild inhomogeneity interpreted as normal or unspecific; 2, several rounded; 3, numerous or confluent hypoechoic lesions) and a score of 2 or 3 indicated typical pSS.

### 2.5 Statistical Analysis

The statistical software IBM SPSS 23.0 (IBM, Armonk, NY, USA) was used for statistical analysis. Descriptive parameters were expressed as the number of patients (%) or means ± standard deviation (SD). Categorical variables were compared with the Chi-Square test. Receiver operating characteristic (ROC) curve analysis and area under the curve (AUC) were performed to determine the accuracy of IC/IVCM for predicting labial gland biopsies, serum antibody status, and pSS classification. Additionally, the absolute agreement, sensitivity, specificity, positive predictive value (PPV), and negative predictive value (NPV) were calculated. The association between IC/ICVM and dry eye/pSS signs was analyzed using Spearman’s correlation coefficients (CCs). An independent *t*-test was used to compare the inflammatory cell density, epidermal and lamina propria thickness, gland density and diameter, OSDI, TMH, and TBUT between the two groups. Logistic regression analyses were used to evaluate the validity of the oral mucosa IC, IVCM, and either of them along with anti-SSA/Ro antibody status compared with labial gland biopsy and ACR-EULAR classification. Bonferroni’s correction was applied to correct for multiple comparisons. *P*-value <0.05 was considered statistically significant.

## 3 Results

### 3.1 Patient Demographics, Dry Eye Signs and ACR-EULAR Criteria

**Figure 2:**
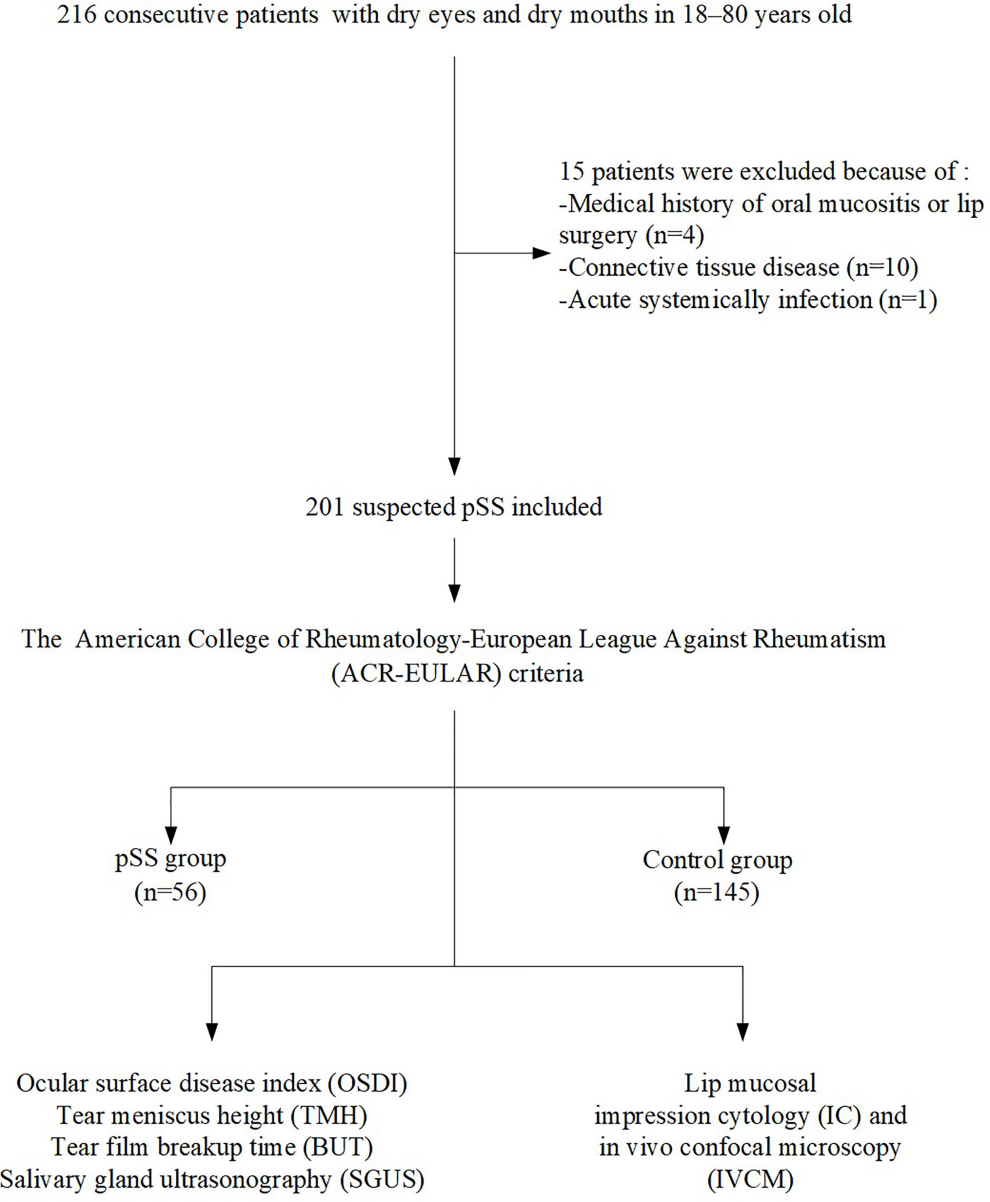
Flowchart of the number of patients with available data per analysis.

A flowchart of inclusion and exclusion criteria and the number of patients included in the analyses are presented in **Figure 2**. A total of 201 individuals (aged 53.32 ± 15.23 years; 162 females) were included and classified according to the ACR-EULAR criteria. The cohort comprised 56 pSS patients (aged 56.07 ± 15.03 years; 54 women) and 145 controls (aged 52.26 ± 15.23 years; 108 women). The age was similar between the two groups (*P* = 0.112); however, a significant difference was detected in gender (*P* = 0.000) as the male:female ratio was 1:9 (1). The OSDI scores in the pSS patients were significantly higher (*P* = 0.000), while the TMH and TBUT values were significantly lower than those observed in the controls (both *P* = 0.000). The demographics, dry eye signs, and ACR-EULAR criteria for the pSS and control groups are shown in **Table 1**. The cost comparison for IC/IVCM with currently used methods is shown in **Table 2**. The IC and IVCM examinations were much less expensive than the labial gland biopsy and serum antibody tests.

**Table 1:** Demographics, dry eye signs, ACR-EULAR criteria, IC and IVCM parameters for patients in the pSS group and control group.

Item	pSS (n = 56)	Control (n = 145)	*P*
**Sex**			
Males, n (%)	2 (3.6%)	37 (25.5%)	0.000*
Females, n (%)	54 (96.4%)	108 (74.5%)	
**Age, year^#^**	56.07 ± 15.03	52.26 ± 15.23	0.112
**Sicca symptom duration, year^$^**	2 (1, 4)	2 (1, 4)	0.072
**OSDI, score^#^**	70.29 ± 27.05	35.95 ± 18.48	0.000*
**TMH, mm^#^**	0.08 ± 0.04	0.15 ± 0.04	0.000*
**TBUT, s^#^**	2.55 ± 1.53	4.06 ± 2.43	0.000*
**ACR-EULAR criteria**			
Focus score of ≥1 for minor labial salivary gland biopsy, n (%)	54 (96.4%)	0 (0%)	0.000*
Presence of anti-SSA antibodies in serum, n (%)	44 (78.6%)	0 (0%)	0.000*
Ocular surface staining score of ≥5, n (%)	40 (71.4%)	22 (15.2%)	0.000*
Schirmer’s test of ≤5 mm/5 min, n (%)	54 (96.4%)	123 (84.8%)	0.000*
Unstimulated whole saliva flow of ≤0.1 ml/min, n (%)	52 (92.9%)	57 (39.3%)	0.000*
Total score^#^	7.96 ± 1.56	1.03 ± 0.58	0.000*
**Other autoantibodies**			
Presence of anti-SSB antibodies in serum, n (%)	18 (32.1%)	0 (0%)	0.000*
Presence of RF in serum, n (%)	20 (35.7%)	0 (0%)	0.000*
Presence of ANA ≥1:320 in serum, n (%)	6 (10.7%)	0 (0%)	0.000*
**IC inflammatory cells density, cells/4 mm^2#^**	52.88 ± 20.71	16.11 ± 7.50	0.000*
**IVCM epithelium layer thickness, μm ^#^**	44.18 ± 23.96	72.09 ± 28.95	0.000*
**IVCM intrinsic layer thickness, μm^#^**	93.26 ± 32.26	194.31 ± 47.71	0.000*
**IVCM labial gland density, cells/mm^2#^**	31.68 ± 8.98	38.50 ± 8.01	0.000*
**IVCM labial gland diameter, μm^#^**	69.76± 22.16	150.13 ± 19.77	0.000*

ACR-EULAR, American College of Rheumatology-European League Against Rheumatism; IC, impression cytology; IVCM, in vivo confocal microscope; pSS, primary Sjögren’s syndrome; OSDI, ocular surface disease index; TMH, tear meniscus height; TBUT, tear film breakup time; anti-SSA, anti-Sjögren’s syndrome A; anti-SSB, anti-Sjögren’s syndrome B; RF, rheumatoid factor; ANA, antinuclear antibody.

^#^mean ± standard deviation; $, median (minimum, maximum).

*P < 0.05.

**Table 2:** A cost comparison for IC/IVCM with currently used methods.

	Cost (dollar)
**New method**	
IC	31.56
IVCM	31.56
**ACR-EULAR criteria**	
Labial gland biopsy	86.32
Serum antibodies	112.04
Ocular surface staining	15.78
Schirmer’s test	0.47
Unstimulated whole saliva flow	0

IC, impression cytology; IVCM, in vivo confocal microscopy.

The cost is only applicable to some hospitals in China.

### 3.2 Lip Mucosal IC Results

#### 3.2.1 Lip Mucosa Morphology

**Figure 3:**
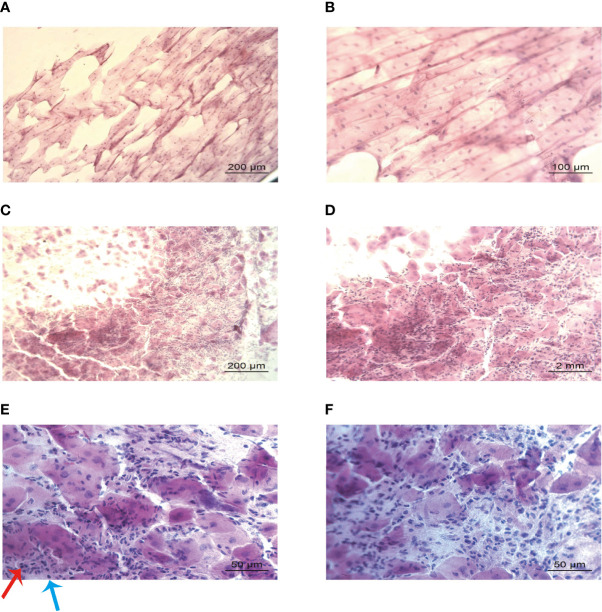
Lip mucosa impression cytology of controls and those with primary Sjögren’s syndrome (H&E). **(A, B)** Large, plump, and organized epithelium without inflammatory infiltration could be seen in the cytology from a control participant: 59-year-old female (A: ×100 and B: ×200). **(C–F)** Shrunken and disorganized epithelium, with massive inflammatory infiltration could be identified in the samples from a 62-year-old female patient with primary Sjögren’s syndrome (**C**: ×100, **D**: ×200, and **(E**, **F)**: ×400). Images E and F showed neutrophils (red arrow) and lymphocytes (blue arrow) infiltration.

**Figure 4:**
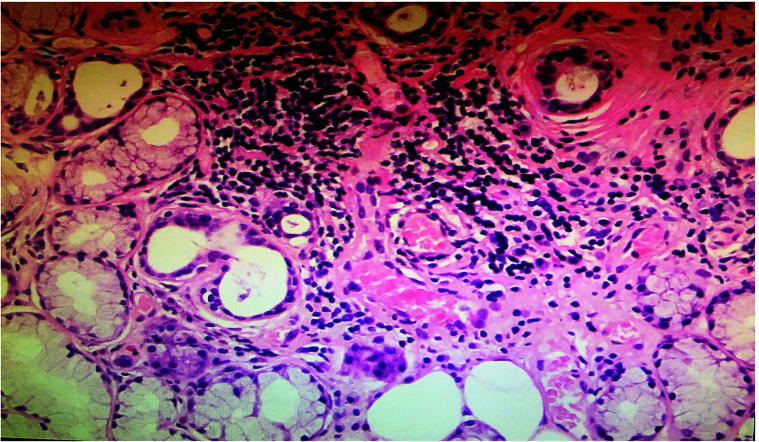
Salivary gland biopsy (H&E). More than 50 infiltrating lymphocytes were observed in each high-power field (magnification, ×200). The biopsy sample was derived from the same primary Sjögren’s syndrome patient (a 62-year-old female) in **Figure 3**.

The IC results from the control group (**Figures 3A, B**) were consistent with the normal histology. The non-keratinized, squamous epithelial cells comprised thin, flat plates that fit closely together with their oval-shaped nuclei (28). No evidence of inflammatory cell infiltration was observed. The H&E staining demonstrated that epithelial cells were smaller in IC samples from patients with pSS than those from controls and had lost their polygonal morphology (**Figures 3C–F**). The cell borders were poorly defined and appeared irregular. The nuclei were elongated and ovoid, with bare nuclei occasionally observed. Massive numbers of inflammatory cells, mainly neutrophils and lymphocytes, were observed in the focal areas (**Figures 3E, F**). Also, abundant inflammatory cells were detected in the same salivary gland biopsy specimen (**Figure 4**). Compared with the controls, the inflammatory cells in the pSS patients were significantly higher (16.11 ± 7.50 vs. 52.88 ± 20.71 cells/4 mm^2^, respectively, *P* = 0.000).

#### 3.2.2 Lip Mucosal IC vs. Labial Gland Biopsy, Anti-SSA/Ro Autoantibody, and ACR-EULAR Classification

Compared with labial gland biopsy results, IC showed an AUC of 0.923 (95% confidence interval (CI): 0.866–0.979) and an optimal cut-off point of 50 cells/4 mm^2^. The absolute agreement between the abnormal IC (≥50 cells/4 mm^2^) and positive labial gland biopsy results (a focus score ≥1) was 95.5% (192/201), with a sensitivity of 85.2% (46/54), specificity of 99.3% (146/147), PPV of 97.9% (46/47), and NPV of 94.8% (146/154).

The IC results also predicted patient anti-SSA/Ro antibody status, with an AUC of 0.854 (95% CI: 0.803–0.925). The absolute agreement between abnormal IC outcomes (≥50 cells/4 mm^2^) and positive anti-SSA/Ro antibody status was 90.5% (182/201), with a sensitivity of 81.8% (36/44), specificity of 93.0% (146/157), PPV of 76.6% (36/47), and NPV of 94.8% (146/154).

Compared to the ACR-EULAR classification, abnormal IC (≥50 cells/4 mm^2^) showed an AUC of 0.907 (95% CI: 0.847–0.968). The absolute agreement between abnormal IC outcomes and ACR-EULAR classification (total score ≥4) was 94.5% (190/201) with a sensitivity of 82.1% (46/56), specificity of 99.3% (144/145), PPV of 97.9% (46/47), and NPV of 93.5% (144/154) (**Table 3**).

**Table 3:** Lip mucosal IC results versus labial gland biopsy, autoantibody, and classification criteria results.

	Labial gland biopsy (n = 201)	Anti-SSA/Ro antibody (n = 201)	ACR-EULAR classification (n = 201)
**% Absolute agreement**	95.5% (192/201)	90.5% (182/201)	94.5% (190/201)
**Sensitivity**	85.2% (46/54)	81.8% (36/44)	82.1% (46/56)
**Specificity**	99.3% (146/147)	93.0% (146/157)	99.3% (144/145)
**PPV**	97.9% (46/47)	76.6% (36/47)	97.9% (46/47)
**NPV**	94.8% (146/154)	94.8% (146/154)	93.5% (144/154)

IC, impression cytology; Anti-SSA, Anti- Sjögren’s syndrome A; ACR-EULAR, American College of Rheumatology-European League Against Rheumatism; PPV, positive predictive value; NPV, negative predictive value.

### 3.3 Analysis of the Lip Mucosal IVCM

#### 3.3.1 Lip Mucosal Morphology by IVCM

**Figure 5:**
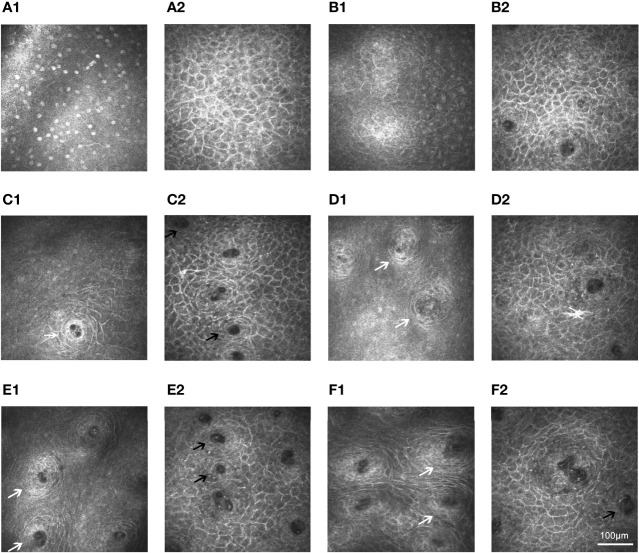
*In vivo* confocal microscopy of lip mucosa in controls and those with primary Sjögren’s syndrome. **(A1–A2)** Superficial layer could be seen in the IVCM images from a 59-year-old female control (**A1**: 26-µm) and a 62-year-old female pSS patient (**A2**: 10-µm). **(B1–B2)** Similar depth (30-µm) renderings of structures in a 55-year-old female control **(B1)** and a 56 -year-old female patient with pSS **(B2)**. **(C1–D2)** In a 59-year-old female control, labial glands (white arrow) appeared at 120-µm level **(C1)** and were obviously at 200-µm depth **(D1)**. However, in a 62-year-old female patient with pSS, the labial glands (black arrow) appeared at 50-µm level **(C2)** and were not clearly visible after the 150-µm depth **(D2)**. **(E1–F1)** Regular circle labial glands (white arrow) at a depth of 255-µm level could be seen from controls: 58-year-old male **(E1)** and 60-year-old female **(F1)**. **(E2–F2)** Small and irregular glands (black arrow) at a depth of 95-µm level could be identified in the images from pSS patients: 57-year-old male **(E2)** and 61-year-old female **(F2)**. The **A1**, **C1**, and **D1** were derived from the same control (a 59-year-old female) in **Figure 3**; the **A2**, **C2**, and **D2** were derived from the same pSS patient (a 62-year-old female) in **Figures 3**, **4**. IVCM, *in vivo* confocal microscopy; pSS, primary Sjögren’s syndrome.

The IVCM results of the control group (**Figures 5A1, B1**) were consistent with the IVCM appearance of normal lip mucosa (16, 17, 29, 30). In patients with pSS, the epithelial cells were thinner and smaller than those in controls and had bright, irregular borders and small, inconspicuous nuclei (**Figures 5A2, B2**). However, the number of epithelial cells was similar between the groups. The mucosa epithelia and intrinsic layers in patients with pSS were atrophied, and fewer and smaller glands were detected than in control individuals (**Table 1**). Furthermore, the thickness of the epithelium (44.18 ± 23.96 μm) and intrinsic layers (93.26 ± 32.26 μm) was significantly thinner than that of the corresponding structures in the control group (72.09 ± 28.95 μm and 194.31 ± 47.71 μm, respectively, both *P* = 0.000). **Figures 5C1–F2** show that the pSS group had significantly lower average gland density (31.68 ± 8.98 cells/mm^2^) and diameters (69.76 ± 22.16 μm) than the control group (38.50 ± 8.01 cells/mm^2^ and 150.13 ± 19.77 μm, respectively, both *P* = 0.000).

#### 3.3.2 Lip Mucosal IVCM vs. Labial GlandBiopsy, Anti-SSA/Ro Autoantibody, and ACR-EULAR Classification

The accuracy of the lip mucosal atrophy and gland density/diameter for predicting the labial gland biopsy, anti-SSA/Ro antibody status, and the ACR-EULAR classification are shown in **Table 4**. The accuracy of the lip mucosal epidermis thickness/labial gland density to predict positive ACR-EULAR classification (total score ≥4) was poor, with an AUC of 0.702 (95% CI: 0.598 to 0.803)/0.654 (95% CI: 0.526 to 0.788), and an optimal cut-off point of 60 μm/29 cells/mm^2^. The accuracy of lamina propria thickness/labial gland diameter to predict positive ACR-EULAR classification (total score ≥4) was good, with an AUC of 0.868 (95% CI: 0.759 to 0.926)/0.906 (95% CI: 0.824 to 0.964), and an optimal cut-off point of 128 μm/114 μm. An intrinsic layer thickness of ≤128 μm was found in 85.7% (48/56) of the patients with pSS but in only 9.7% (14/145) of the controls, showing an absolute agreement of 89.1% (179/201), a sensitivity of 85.7% (48/56), a specificity of 90.3% (131/145), a PPV of 77.4% (48/62), and an NPV of 94.2% (131/139) A gland diameter ≤114 μm was found in 89.3% (50/56) of the patients with pSS but in only 4.1% (6/145) of the controls; the absolute agreement was 94.0% (189/201), with a sensitivity of 89.3% (50/56), a specificity of 95.9% (139/145), a PPV of 89.3% (50/56), and an NPV of 95.9% (139/145).

**Table 4:** Lip mucosal IVCM results as compared with labial gland biopsy, autoantibody, and classification criteria results.

	Labial gland biopsy			
	Epithelial layer ≤60 μm	Intrinsic layer ≤128 μm	Gland density ≤29 cells/mm^2^	Gland diameter ≤114 μm
**Absolute agreement**	71.6% (144/201)	90.0% (181/201)	79.6% (160/201)	93.0% (187/201)
**Sensitivity**	77.8% (42/54)	88.9% (48/54)	44.4% (24/54)	88.9% (48/54)
**Specificity**	69.4% (102/147)	90.5% (133/147)	92.5% (136/147)	94.6% (139/147)
**PPV**	48.3 (42/87)	77.4% (48/62)	66.6% (24/35)	85.7% (48/56)
**NPV**	89.5 (102/114)	95.7% (133/139)	81.9% (136/166)	95.9% (139/145)
	**Anti-SSA/Ro antibody**			
	**Epithelial layer ≤60 μm**	**Intrinsic layer ≤128 μm**	**Gland density ≤29 cells/mm^2^**	**Gland diameter ≤114 μm**
**Absolute agreement**	70.6% (142/201)	85.1% (171/201)	80.6% (162/201)	90.0% (181/201)
**Sensitivity**	81.8% (36/22)	86.4% (38/44)	45.5% (20/44)	90.9% (40/44)
**Specificity**	67.5% (106/157)	84.7% (133/157)	90.4% (142/157)	89.8% (141/157)
**PPV**	41.4% (36/87)	61.3% (38/62)	57.1% (20/35)	71.4% (40/56)
**NPV**	93.0% (106/114)	95.7% (133/139)	85.5% (142/166)	97.2% (141/145)
	**ACR-EULAR classification**			
	**Epithelial layer ≤60 μm**	**Intrinsic layer ≤128 μm**	**Gland density ≤29 cells/mm^2^**	**Gland diameter ≤114 μm**
**Absolute agreement**	70.6% (142/201)	89.1% (179/201)	78.6% (158/201)	94.0% (189/201)
**Sensitivity**	75% (42/56)	85.7% (48/56)	42.9% (24/56)	89.3% (50/56)
**Specificity**	69.0% (100/145)	90.3% (131/145)	92.4% (134/145)	95.9% (139/145)
**PPV**	48.3% (42/87)	77.4% (48/62)	68.6% (24/35)	89.3% (50/56)
**NPV**	87.7% (100/114)	94.2% (131/139)	80.7% (134/166)	95.9% (139/145)

IVCM, in vivo confocal microscopy; Anti-SSA, anti-Sjögren’s syndrome A; ACR-EULAR, American College of Rheumatology-European League Against Rheumatism; PPV, positive-predictive value; NPV, negative-predictive value.

### 3.4 Predictive Value of the Combination of IC/IVCM and Anti-SSA/Ro Antibody Status

The predictive value of the combined IC/IVCM values and anti-SSA/Ro antibody status is shown in **Table 5**. In patients with positive IC (≥50 cells/4 mm^2^)/anti-SSA/Ro antibodies, 96.3% (52/54) had positive labial gland biopsy results (a focus score ≥1) and 96.4% (54/56) fulfilled the ACR-EULAR criteria (total score ≥4). In patients with negative IC results (<50 cells/4 mm^2^) and without anti-SSA/Ro antibodies, 98.0% (144/147) had negative labial gland biopsy results (a focus score <1), while 99.3% (144/145) did not meet the ACR-EULAR criteria (total score <4).

**Table 5:** Predictive value of the combination of IC/IVCM and antibody status.

	Positive IC with anti SSA antibodies	
	Labial gland biopsy	ACR-EULAR classification
**Absolute agreement**	97.5% (196/201)	98.5% (198/201)
**Sensitivity**	96.3% (52/54)	96.4% (54/56)
**Specificity**	98.0% (144/147)	99.3% (144/145)
**PPV**	94.5% (52/55)	98.2% (54/55)
**NPV**	98.6% (144/146)	98.6% (144/146)
	**Intrinsic layer ≤128 μm with anti SSA antibodies**	
	**Labial gland biopsy**	**ACR-EULAR classification**
**Absolute agreement**	91.0% (183/201)	92.0% (185/201)
**Sensitivity**	96.3% (52/54)	96.4% (54/56)
**Specificity**	89.1% (131/147)	90.3% (131/145)
**PPV**	76.5% (52/68)	79.4% (54/68)
**NPV**	98.5% (131/133)	98.5% (131/133)
	**Gland diameter ≤114 μm with anti SSA antibodies**	
	**Labial gland biopsy**	**ACR-EULAR classification**
**Absolute agreement**	95.0% (191/201)	96.0% (193/201)
**Sensitivity**	96.3% (52/54)	96.4% (54/56)
**Specificity**	94.6% (139/147)	95.7% (139/145)
**PPV**	86.7% (52/60)	90% (54/60)
**NPV**	98.6% (139/141)	98.6% (139/141)

IC, impression cytology; IVCM, in vivo confocal microscopy; Anti-SSA, Anti-Sjögren’s syndrome A; ACR-EULAR, American College of Rheumatology-European League Against Rheumatism; PPV, positive predictive value; NPV, negative predictive value.

In patients with intrinsic layer thicknesses of ≤128 μm or positive anti-SSA/Ro antibodies, 96.3% (52/54) had positive labial gland biopsy results (a focus score ≥1) and 96.4% (54/56) fulfilled the ACR-EULAR criteria (total score ≥4) with a specificity of 89.1% (131/147) and 90.3% (131/145), respectively. In patients with gland diameters ≤114 μm or positive anti-SSA/Ro antibodies, 96.3% (52/54) had positive labial gland biopsies (a focus score ≥1) and 96.4% (54/56) fulfilled the ACR-EULAR criteria (total score ≥4). In patients with gland diameters >114 μm and without anti-SSA/Ro antibodies, 94.6% (139/147) had negative labial gland biopsies (a focus score <1) and 95.7% (139/145) did not fulfill the ACR-EULAR criteria (total score <4).

In patients with positive labial gland biopsies (a focus score ≥1) and negative anti-SSA/Ro antibodies (n = 2), both of them presented normal oral mucosa IC (<50 cells/4 mm^2^) and some IVCM results, including epidermis thickness >60 μm, lamina propria thickness >128 μm, and labial gland density >29 cells/mm^2^, however, abnormal gland diameter (≤114 μm). There was an abnormal oral mucosa IC (≥50 cells/4 mm^2^) and lamina propria thickness ≤128 μm in 83.3% (10/12) of patients with positive anti-SSA/Ro antibodies but negative labial gland biopsies (n = 12), 50% (6/12) had epidermis thickness ≤60 μm, 33.3% (4/12) had labial gland density ≤29 cells/mm^2^, and 100% had gland diameter ≤114 μm.

### 3.5 Lip Mucosal IC and IVCM Results as Compared With Anti-SSB antibody, RF and ANA Status

The accuracy of the lip mucosal IC and IVCM for predicting the anti-SSB antibody, RF and ANA status is shown in **Table 6**. Abnormal IC outcomes (≥50 cells/4 mm^2^) were found in 83.3% (15/18) of patients with positive anti-SSB antibodies, 90% (18/20) with positive RF, and 83.3% (5/6) with positive ANA, with specificities of 82.5% (151/183), 84.0% (152/181), 78.5% (153/195), respectively. Intrinsic layer thicknesses of ≤128 μm were found in 88.9% (16/18) of patients with positive anti-SSB antibodies, 90% (18/20) with positive RF, and 66.7% (4/6) with positive ANA, with specificities of 74.9% (137/183), 75.7% (137/181), 70.3% (137/195), respectively. Gland diameters ≤114 μm were found in 77.8% (14/18) of patients with positive anti-SSB antibodies, 90% (18/20) with positive RF, and 83.3% (5/6) with positive ANA, with specificities of 77% (141/183), 79% (143/181), and 73.8% (144/195), respectively.

**Table 6:** Lip mucosal IC and IVCM results as compared with anti-SSB antibody, RF and ANA.

	Anti-SSB antibody				
	IC ≥50 cells/4 mm^2^	Epithelial layer ≤60 μm	Intrinsic layer ≤128 μm	Gland density ≤29 cells/mm^2^	Gland diameter ≤114 μm
**Absolute agreement**	82.6% (166/201)	61.7% (124/201)	76.1% (153/201)	79.6% (160/201)	77.1% (155/201)
**Sensitivity**	83.3% (15/18)	77.8% (14/18)	88.9% (16/18)	33.3% (6/18)	77.8% (14/18)
**Specificity**	82.5% (151/183)	60.1% (110/183)	74.9% (137/183)	84.2% (154/183)	77.0% (141/183)
**PPV**	68.1% (15/47)	16.1% (14/87)	25.8% (16/62)	17.1% (6/35)	25.0% (14/56)
**NPV**	98.1% (151/154)	96.5% (110/114)	98.6% (137/139)	92.8% (154/166)	97.2% (141/145)
	**Rheumatoid Factor**				
	**IC ≥50 cells/4 mm^2^**	**Epithelial layer ≤60 μm**	**Intrinsic layer ≤128 μm**	**Gland density ≤29 cells/mm^2^**	**Gland diameter ≤114 μm**
**Absolute agreement**	84.6% (170/201)	62.7% (126/201)	77.1% (155/201)	76.6% (154/201)	80.1% (161/201)
**Sensitivity**	90.0% (18/20)	80.0% (16/20)	90.0% (18/20)	20.0% (4/20)	90.0% (18/20)
**Specificity**	84.0% (152/181)	60.8% (110/181)	75.7% (137/181)	82.9% (150/181)	79.0% (143/181)
**PPV**	38.3% (18/47)	18.4% (16/87)	29.0% (18/62)	11.4% (4/35)	32.1% (18/56)
**NPV**	98.7% (152/154)	96.5% (110/114)	98.6% (137/139)	90.4% (150/166)	98.6% (143/145)
	**Antinuclear Antibody**				
	**IC ≥50 cells/4 mm^2^**	**Epithelial layer ≤60 μm**	**Intrinsic layer ≤128 μm**	**Gland density ≤29 cells/mm^2^**	**Gland diameter ≤114 μm**
**Absolute agreement**	78.6% (158/201)	58.7% (118/201)	80.1% (141/201)	83.6% (168/201)	74.1% (149/201)
**Sensitivity**	83.3% (5/6)	83.3% (5/6)	66.7% (4/6)	33.3% (2/6)	83.3% (5/6)
**Specificity**	78.5% (153/195)	57.9% (113/195)	70.3% (137/195)	83.1% (162/195)	73.8% (144/195)
**PPV**	10.6% (5/47)	5.7% (5/87)	6.5% (4/62)	5.7% (2/35)	8.9% (5/56)
**NPV**	99.4% (153/154)	99.1% (113/114)	98.6% (137/139)	97.6% (162/166)	99.3% (144/145)

IC, impression cytology; IVCM, in vivo confocal microscopy; Anti-SSB, anti-Sjögren’s syndrome B; RF, rheumatoid factor; ANA, antinuclear antibody; PPV, positive-predictive value; NPV, negative-predictive value.

### 3.6 Analysis of SGUS Scores

Hypoechoic lesions (score 2 or 3), typical of pSS, were found in 57.1% (32/56) of pSS patients with a specificity of 95.9% (139/145). The accuracy of abnormal SGUS in predicting labial gland biopsy results, anti-SSA/Ro antibody status, and ACR-EULAR classification is shown in **Table 7**.

**Table 7:** Abnormal salivary gland ultrasound results compared with labial gland biopsy, anti-SSA/Ro antibody status, and ACR-EULAR classification results.

	Labial gland biopsy (n = 201)	Anti-SSA/Ro antibody (n = 201)	ACR-EULAR classification (n = 201)
**Absolute agreement**	84.1% (169/201)	87.1% (175/201)	85.1% (171/201)
**Sensitivity**	55.6% (30/54)	63.3% (28/44)	57.1% (32/56)
**Specificity**	94.6% (139/147)	93.6% (147/157)	95.9% (139/145)
**PPV**	78.9% (30/38)	73.7% (28/38)	84.2% (32/38)
**NPV**	85.3% (139/163)	90.2% (147/163)	85.3% (139/163)

Anti-SSA, Anti-Sjögren’s syndrome A; ACR-EULAR, American College of Rheumatology-European League Against Rheumatism; PPV, positive predictive value; NPV, negative predictive value.

### 3.7 Associations Between Lip Mucosal IC/ICVM and Dry Eye/pSS Signs

The correlations between the IC/ICVM results and dry eye/pSS signs are shown in **Table 8**. The IC results showed good correlations with anti-SSA status (CC = 0.743), labial gland biopsy (CC = 0.899), and ACR-EULAR criteria (CC = 0.738) (all *P* = 0.000). Lamina propria thickness and gland diameter also have marked correlations with anti-SSA status (CC = 0.614, CC = 0.663), labial gland biopsy (CC = 0.701, CC = 0.741), and ACR-EULAR criteria (CC = 0.600, CC = 0.683) (all *P* = 0.000). There were also good correlations between UWS and IC (CC = 0.687), lamina propria thickness (CC = 0.566), and gland diameter (CC = 0.543) (all *P* = 0.000). The SGUS score also showed marked correlations with IC (CC = 0.469) and gland diameter (CC = 0.429) (all *P* = 0.000). Dry eye symptoms and signs also showed significant associations with IC/IVCM results (**Table 8**).

**Table 8:** Correlation between lip mucosal IC/ICVM and dry eye/pSS signs.

		IC	Epithelial layer thickness	Lamina propria thickness	Gland density	Gland diameter
**Anti-SSA/Ro**	CC	0.743^**^	0.426^**^	0.614^**^	0.316^**^	0.663^**^
	*P*	0.000	0.000	0.000	0.000	0.000
**Labial gland biopsy**	CC	0.899^**^	0.472^**^	0.701^**^	0.303^**^	0.741^**^
	*P*	0.000	0.000	0.000	0.000	0.000
**ST**	CC	0.452^**^	0.139^*^	0.316^**^	0.133	0.527^**^
	*P*	0.000	0.049	0.000	0.060	0.000
**OSS**	CC	0.472^**^	−0.353^**^	−0.392^**^	−0.090	−0.397^**^
	*P*	0.000	0.000	0.000	0.206	0.000
**UWS**	CC	0.687^**^	0.513^**^	0.566^**^	0.162^*^	0.543^**^
	*P*	0.000	0.000	0.000	0.022	0.000
**ACR-EULAR criteria**	CC	0.738^**^	0.418^**^	0.600^**^	0.198^**^	0.683^**^
	*P*	0.000	0.000	0.000	0.000	0.000
**OSDI**	CC	0.514^**^	−0.348^**^	−0.442^**^	−0.050	−0.449^**^
	*P*	0.000	0.000	0.000	0.477	0.000
**TMH**	CC	0.553^**^	0.291^**^	0.449^**^	0.172^*^	0.522^**^
	*P*	0.000	0.000	0.000	0.014	0.000
**TBUT**	CC	0.294^**^	0.083	0.300^**^	0.142^*^	0.305^**^
	*P*	0.000	0.240	0.000	0.045	0.000
**SGUS**	CC	0.469^**^	−0.357^**^	−0.394^**^	−0.256^**^	−0.429^**^
	*P*	0.000	0.000	0.000	0.000	0.000

IC, impression cytology; IVCM, in vivo confocal microscopy; pSS, primary Sjögren’s syndrome; Anti-SSA/Ro, Anti-Sjögren’s syndrome A/Ro; ST, Schirmer test; OSS, ocular staining score; UWS, unstimulated whole saliva flow; ACR-EULAR, American College of Rheumatology-European League Against Rheumatism; OSDI, ocular surface disease index; TMH, tear meniscus height; TBUT, tear breakup time; SGUS, salivary gland ultrasonography; CC, correlation coefficient.

The correlation coefficient is shown in bold for all significant correlations (P < 0.05).

*P < 0.01; **P < 0.001.

**Figure 6:**
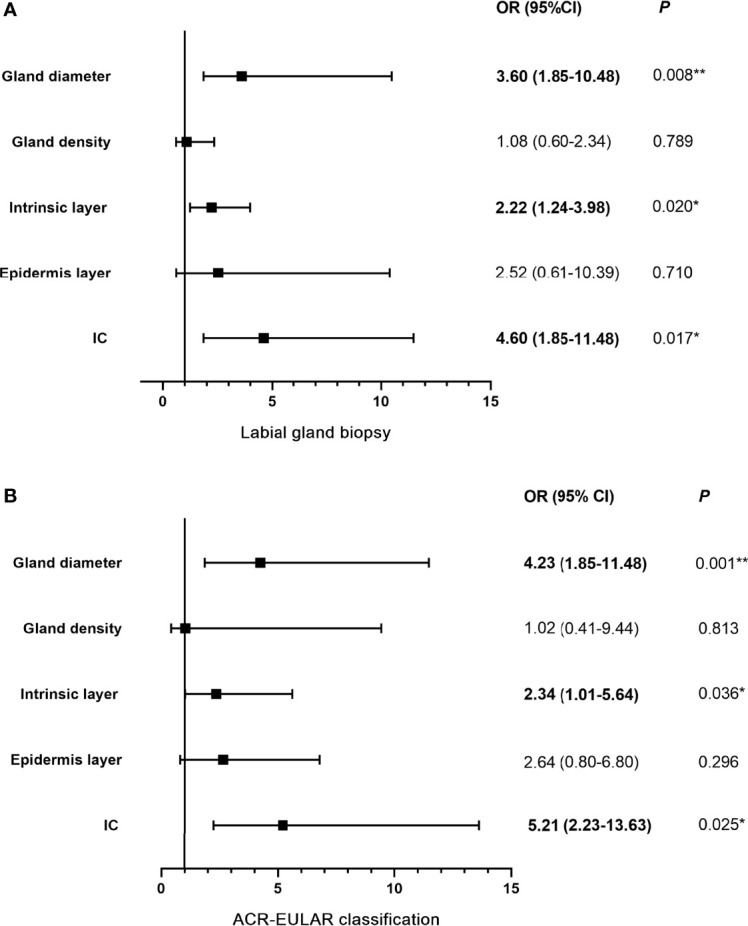
Logistic regression models: associations among lip mucosa IC/IVCM and labial gland biopsy **(A)** and ACR-EULAR classification **(B)**. OR (95% CI) indicates the risk of positive labial gland biopsy and ACR-EULAR classification enhanced when IC ≥50 cells/4 mm^2^, epithelial layer ≤60 μm, intrinsic layer ≤128 μm, gland density ≤29 cells/mm^2^ and gland diameter ≤114 μm. IC, impression cytology; IVCM, *in vivo* confocal microscope; ACR-EULAR, American College of Rheumatology-European League Against Rheumatism; OR, odds ratio; CI: confidence interval; *P < 0.05; **P < 0.01.

The associations between lip mucosa IC/IVCM results and labial gland biopsy and ACR-EULAR classification are shown in **Figure 6**. After multivariable adjustment, participants with abnormal IC results (≥50 cells/4 mm^2^) showed an OR 4.60 (95% CI: 1.85–11.48) for positive labial gland biopsy (a focus score ≥1) and OR 5.21 (95% CI: 2.23–13.63) for ACR-EULAR classification (total score ≥4) than those with normal IC results. Patients with intrinsic layer thicknesses of ≤128 μm had OR 2.22 (95% CI: 1.24–3.98) for positive labial gland biopsy and OR 2.34 (95% CI: 1.01–5.64) for ACR-EULAR classification than those with intrinsic layer thicknesses >128 μm. Participants with labial gland diameters ≤114 μm presented an OR 3.60 (95% CI: 1.85–10.48) for positive labial gland biopsy and OR 4.23 (95% CI: 1.85–11.48) for ACR-EULAR classification than those with gland diameters >114 μm. No significant association of epithelium layer thickness/labial gland density with labial gland biopsy or ACR-EULAR classification was observed. Because of collinearity, the IC/IVCM in combination with anti-SSA/Ro status was not included in the logistic analysis models.

## 4 Discussion

To date, a consensus does not exist regarding the classification criteria for patients with suspected pSS. The labial gland biopsy findings are most strongly relevant, but the procedure has limited application because of the associated patient discomfort and fear (4, 5, 31). Thus, new, non-invasive or mini-invasive classification measures are needed. This study assessed the lip mucosal IC and IVCM findings in a representative population of patients with clinically suspected pSS. We compared the validity of these examinations with labial gland biopsy results, anti-SSA/Ro antibody status, and the ACR-EULAR criteria.

Lip mucosal IC and labial gland biopsy are both histopathological examinations. The labial gland biopsy examines inflammatory cell infiltration into the glands and surrounding tissues of the lip mucosa propria layer (2, 9). IC removes the first to third most superficial layers (almost 5–15 μm) (26), and deeper tissue cells may be obtained if multiple IC samples are taken from the same position. In this study, the same regions were sampled thrice and IC detected massive inflammatory cell infiltration into the lip mucosa deep epithelial tissue (at a depth of 15–45 μm) of patients with pSS, unlike in samples obtained from controls. The typical IC finding (≥50 inflammatory cells/4 mm^2^) was positively correlated with labial gland biopsy results and ACR-EULAR criteria, with excellent correlation coefficients of 0.899 and 0.738, respectively. This correlation indicated that a patient with a typical IC was likely to show a positive labial gland biopsy result and be classified as pSS. Currently, pathological changes associated with pSS focus mainly on the infiltration of inflammatory cells into the labial glands within the lamina propria (2, 9, 10). However, the presence of epithelial inflammatory cell infiltration captured by IC samples might be another pathognomonic feature of pSS. Moreover, in lip mucosal IVCM examinations, we found that the lip mucosal epithelium and lamina propria of patients with pSS had atrophied, which conformed to the pathophysiological process of the disease (1, 2), resulting in shallow glandular structures. Therefore, repeated sampling at the same location may obtain a deep layer or even periglandular tissue. Consequently, the evidence of the infiltration of inflammatory cells in deep tissue (almost 15–45 μm) observed by lip mucosa IC examination was similar to the labial gland biopsy results. Additionally, IC showed good absolute agreement (95.5%), sensitivity (85.2%), and specificity (99.3%) with the labial gland biopsy results. Importantly, the IC results accurately predicted the positive and negative results of labial gland biopsy (PPV 97.9 and NPV 94.8%, respectively).

These findings also showed that positive IC results had good absolute agreement (94.5%), sensitivity (82.1%), and specificity (99.3%) with the ACR-EULAR classification criteria. The PPV (97.9%) was higher than the NPV (93.5%), indicating that a positive IC result predicts the fulfillment of the ACR-EULAR criteria, but a negative IC does not exclude the possible fulfillment of the criteria. Negative serology is well-established to occur in 10–50% of patients with pSS (9, 32–34). In this study, 21.4% (12/56) of the patients with pSS were negative for anti-SSA antibodies. When labial gland biopsies were considered, all patients without anti-SSA/Ro antibodies were classified as pSS. Additionally, in these anti-SSA/Ro-negative patients, positive biopsies were decisive for the classification according to ACR-EULAR criteria (2, 9). However, when lip mucosal IC was considered a replacement for labial gland biopsies, 83.3% (10/12) of our patients without anti-SSA/Ro antibodies were classified as pSS, demonstrating the potential of IC as a substitute for salivary gland biopsy. Furthermore, the predictive value increased when combining the IC result and anti-SSA/Ro antibody status. However, lip mucosa IC cannot fully replace labial gland biopsies since the serologically negative patients were underdiagnosed. Consequently, lip mucosa IC might be a recommended tool in patients without anti-SSA/Ro antibodies, especially when the gland function is impaired. However, labial gland biopsy might remain the final consideration for diagnosing pSS in the absence of anti-SSA/Ro antibodies and a negative IC.

The superficial layer of lip mucosa could be affected by a variety of factors, such as oral mucositis and salivary glands, which also cause superficial inflammatory cell infiltration (9, 10). However, we excluded patients with recent oral mucositis and infections, and no such clinical manifestations were found during patient examinations. Despite limitations, lip mucosal IC was proposed as a new pathological method for classifying pSS and as a possible biopsy replacement because of the high diagnostic value and correlations with labial gland biopsy results and ACR-EULAR criteria.

The atrophy of the lip mucosa and decreased gland density/diameter were evident in the IVCM results of patients with pSS. A large number of patients with IVCM-determined thin lip mucosal thicknesses or small gland diameters had positive labial gland biopsy results and fulfilled the ACR-EULAR criteria. Lamina propria atrophy was obvious in pSS patients, which was consistent with the pathophysiological changes associated with the disease, and inflammatory cell infiltration occurred around the labial glands and surrounding tissues (1–3). When combined with intrinsic mucosal depth of ≤128 μm/gland diameter ≤114 μm and positive antibody status, the prediction of positive labial gland biopsy and ACR-EULAR classification increased (>96%). These findings demonstrate the high predictive value of combining IVCM results with anti-SSA/Ro antibody status. Moreover, logistic regression analyses also identified that IVCM might be a promising predictive tool for pSS.

Recently, SGUS has become a recommended, non-invasive diagnostic methodology for patients with presumptive pSS (14). A recent meta-analysis assessing the diagnostic properties of SGUS in pSS reported a sensitivity of 69% and a specificity of 92% (35). The current findings were similar to the above results; SGUS is highly specific for pSS, but the sensitivity is poor. In this study, lip mucosal IC and IVCM showed higher diagnostic properties than SGUS. Strikingly, more than 96% patients with a positive IC/IVCM result and anti-SSA/Ro antibody fulfilled the ACR-EULAR criteria. Moreover, the results of these methods showed close associations with ACR-EULAR criteria, suggesting that changes in the lip mucosa are related to disease pathogenesis. Many studies have clearly indicated that salivary gland epithelial cells are active in pSS progression, both as targets and conductors of immune response (36, 37). The epithelial cells can act as non-professional antigen presenting cells and play key roles in recruitment and activation of immune cells as well as the activation and differentiation of T and B cells (36, 37). SSA/SSB proteins mainly reside in the nucleus or cytoplasm of the epithelial cell (37). However, these proteins can be seen by the immune system through some mechanisms and produce autoantibodies (37), even in the conjunctival epithelial cells collected by IC (38). Studies have shown that salivary gland epithelial cells are an active site of autoantigen synthesis (36, 37). Those epithelial cell ultrastructure alterations could not be captured by SGUS, but might be captured by IC/IVCM. Therefore, lip mucosal IC and IVCM are strong candidates as non-invasive predictive methods to assess the involvement of salivary glands in pSS patients.

Here, we have shown the benefit of applying IC and IVCM to reveal lip mucosa morphological pathology in patients with presumptive pSS. These techniques have the advantage of non-invasive evaluation that might be included in regular outpatient visits during repeated follow-ups. A major advantage of IC is that the cells are arranged naturally, enabling clinical pathologists to examine an exact copy of the area of interest (25). In addition, IVCM is also a non-invasive, time-saving, real-time imaging technique that facilitates accurate clinical assessments and regular follow-ups without any risk to the patient (16). IC and IVCM may also facilitate patient stratification, early detection, and treatment in appropriate departments. IC and IVCM are routinely available and performed in the department of ophthalmology. However, a huge proportion of SS patients are not diagnosed by an ocular specialist, but rather by a rheumatologist, a primary care or internal medicine physician, or an oral specialist. The IC and IVCM may not be easily accessible in all settings, and not all eye specialists are able to evaluate the oral mucosa in addition to the ocular surface. Therefore, standardized training is necessary.

Further elucidation of the roles of IC and IVCM in the diagnosis of pSS is essential. First, larger groups should be included to refine the specificity and sensitivity of the methods. Second, the development of an international consensual scoring system for lip mucosal IC/IVCM is required to discover the underlying connective tissue diseases, early-stage performance, evaluate disease severity (such as lymphoma), as well as increase intra- and inter-observer reproducibility. Third, longitudinal studies are warranted to correlate the glandular inflammatory cell infiltration and damage with IC and the IVCM results and assess patient outcomes in response to therapy. Fourth, this study did not look at secondary Sjögren’s syndrome and other disease controls (such as rheumatoid arthritis, systemic lupus erythematosus, diabetes, etc.), and healthy controls. These need to be clarified in future studies.

In conclusion, the lip mucosal IC and IVCM provided highly specific and accurate assessments of the pathological changes in pSS patients. These techniques provide a simplified diagnostic process that is suitable for inclusion within a modified classification system that might serve as a starting point for the non-invasive diagnosis of pSS.

## Data Availability Statement

The raw data supporting the conclusions of this article will be made available by the authors, without undue reservation.

## Ethics Statement

The studies involving human participants were reviewed and approved by the Human Research and Ethics Committee of Peking University Third Hospital (No. M2019236). The patients/participants provided their written informed consent to participate in this study.

## Author Contributions

RH, YC, and XR setup the protocol and recruited the participants. RH collected and analyzed the data, created the figures, and contributed to the writing of the manuscript. ZL discussed the data and participated in writing the manuscript. YW provided statistical advice and oversaw the statistical analysis. XJ provided advice on the protocol and statistical analysis. XL setup the protocol, supervised the study, and wrote the final manuscript. All authors listed have made a substantial, direct, and intellectual contribution to the work and approved it for publication.

## Funding

This work was supported by the Capital’s Funds for Health Improvement and Research [grant numbers: CFH2018-2-4093]. The funding organization had no role in the design or conduct of this research.

## Conflict of Interest

The authors declare that the research was conducted in the absence of any commercial or financial relationships that could be construed as a potential conflict of interest.

## Publisher’s Note

All claims expressed in this article are solely those of the authors and do not necessarily represent those of their affiliated organizations, or those of the publisher, the editors and the reviewers. Any product that may be evaluated in this article, or claim that may be made by its manufacturer, is not guaranteed or endorsed by the publisher.
